# The Impact of Image-Guided Screw Placement on Unexpected Return to the Operating Room: A Systematic Review and Meta-Analysis of 91,285 Patients

**DOI:** 10.1177/21925682261493032

**Published:** 2026-09-28

**Authors:** Jaskaran Singh, Marion Sylvain, Mikko Vorster, Bona Baissa, Aazad Abbas, Michael Goytan

**Affiliations:** 1 Division of Orthopaedic Surgery, 12359 University of Manitoba, Winnipeg, MB, Canada; 2 Faculty of Medicine and Health Sciences, 12367 McGill University, Montreal, QC, Canada; 3 Division of Orthopaedic Surgery, 7938 University of Toronto, Toronto, ON, Canada; 4 Holland Bone & Joint Program, Sunnybrook Research Institute, Toronto, Canada; 5 Institute of Biomedical Engineering, 7938 University of Toronto, Toronto, ON, Canada

**Keywords:** navigation, reoperation, pedicle, screw, spine, III

## Abstract

**Study design:**

Systematic Review and Meta-Analysis.

**Objectives:**

Navigation-guided screw placement improves screw accuracy and may reduce hardware-related revision, yet its effect on overall unexpected return to the operating room (URTO) remains uncertain. This study compares image-guided (navigation and robotics) with freehand screw placement for hardware-related revision, non-hardware reoperation, and URTO following spinal fixation.

**Methods:**

A PRISMA-guided systematic review identified comparative studies evaluating image-guided versus freehand screw fixation. Two reviewers performed screening, data extraction, and risk-of-bias assessment. Odds ratios (ORs) were pooled using a random-effects Mantel-Haenszel meta-analysis. Subgroup analyses compared navigated, robotic, and freehand techniques.

**Results:**

Twenty-one studies were included, comprising 91,285 patients (8,743 image-guided; 82,442 freehand). Compared with freehand, image guidance was associated with lower odds of hardware-related revision (OR 0.55, 95% CI 0.40–0.75) and lower odds of URTO (OR 0.69, 95% CI 0.50–0.94), with no difference in non-hardware reoperation (OR 1.03, 95% CI 0.67–1.57). Navigation was associated with lower odds of hardware-related revision (OR 0.51, 95% CI 0.35–0.77) and URTO (OR 0.63, 95% CI 0.51–0.77) versus freehand, whereas robotics showed no significant differences across outcomes. Indirect comparison between navigation and robotics was not statistically significant. Risk of bias was overall high because of retrospective study design, confounding, and inconsistent outcome reporting.

**Conclusions:**

Navigation was associated with lower odds of hardware-related revision and lower odds of URTO, without a difference in non-hardware reoperation. Robotics showed no statistically significant advantage over freehand fixation, but its underrepresentation and differing case mix limit comparative conclusions.

## 1. Introduction

Lateral mass and pedicle screws are widely used in spine surgery to provide stability, fixation, and alignment across diverse pathologies, including instability, deformity, trauma, and degenerative disease. Acting as anchors for fusion constructs, these implants help maintain alignment during osseous healing and have been shown to be safe and effective, making internal screw fixation a cornerstone of modern spinal surgery.^1^

Pedicle screw fixation was originally developed using freehand techniques based on anatomic landmarks, with early descriptions emerging from European orthopaedic surgeons in the mid-20th century, including the foundational work of Boucher,^2^ Roy-Camille,^3^ and Magerl.^4^ Later, frameless stereotactic systems were introduced to augment screw placement accuracy, as described by Kalfas et al in 1995.^5^ With the advancement in intraoperative imaging, CT-based navigation entered routine practice in the early 2000s, giving rise to navigated screw fixation.^6^ In parallel, the adoption of minimally invasive and percutaneous approaches expanded, motivated by the potential to reduce surgical morbidity.^7^

An unexpected return to the operating room (URTO), whether for revision of mispositioned hardware or for reoperation due to other complications, remains a costly and burdensome outcome for patients and health systems.^8^ Prior reviews have reported that navigation reduces hardware-related revision, whereas others find no difference between navigated and freehand techniques, leaving uncertainty about clinical impact.^9,10^ Despite the potential benefits of navigation, there are some studies that have raised concern for non-hardware-related complications and caution that these risks must be interpreted alongside any possible benefits.^11,12^

Given the growing clinical adoption of navigation and robotics and the importance of URTO as a patient-centered outcome, a focused evaluation is warranted. The objective of this study was to compare image-guided screw placement—including navigation and robotics—with freehand fixation with respect to hardware-related revision, non-hardware reoperation, and URTO, with subgroup analyses evaluating navigation and robotics separately.

## 2. Materials and methods

This systematic review was conducted in accordance with the 2020 PRISMA guidelines.^13^ We included human studies comparing image-guided (navigation or robotic) versus conventional freehand/fluoroscopy-guided lateral mass or pedicle screw fixation across the cervical, thoracic, lumbar, or sacral spine. Primary outcomes were unexpected return to the operating room (URTO), hardware-related revision, and non-hardware reoperation.

### 2.1. Search Strategy and Study Selection

MEDLINE (Ovid), Embase (Ovid), and PubMed were searched from inception to March 31st, 2025 using predefined terms related to spine surgery, navigation, robotics, and freehand screw fixation. Citations were screened independently by two reviewers using Covidence, with disagreements resolved by consensus or a third reviewer.

### 2.2. Eligibility Criteria

Eligible studies included randomized controlled trials, prospective and retrospective cohort studies, and case-control studies (Level I–III evidence) reporting navigated or robotic-assisted versus freehand screw fixation and at least one outcome of interest. Exclusions included non-human studies, case reports/series, reviews, conference abstracts without full text, non-English studies, and follow-up <1 month.

### 2.3. Data Extraction and Quality Assessment

Data extraction was performed independently in duplicate, including study characteristics, patient demographics, surgical details (region, indication, guidance method, system used, primary vs. revision, open vs. percutaneous), and outcomes (hardware-related revision, non-hardware reoperation, and URTO). Risk of bias was assessed using RoB 2 for randomized trials and ROBINS-I for non-randomized studies.

### 2.4. Statistical analysis

Binary outcomes were pooled as odds ratios (ORs) with 95% confidence intervals using random-effects models. Heterogeneity was assessed with I^2^. Preplanned subgroup analyses compared navigation versus freehand fixation and robotics versus freehand fixation. An indirect Bucher comparison between navigation and robotics was performed using freehand fixation as the common comparator. Because navigation and robotics were evaluated in substantially different patient populations and surgical indications, this indirect comparison was considered exploratory and susceptible to between-study confounding. Analyses were performed in RStudio using the meta package, with p < 0.05 considered statistically significant.

## 3. Results

### 3.1. Study Characteristics

Of 7601 identified articles, 21 studies^12,14-33^ met inclusion criteria after screening (Figure 1), comprising 19 retrospective cohorts and two randomized trials (Supplemental Table S1). There was considerable agreement during the title and abstract (k = 0.752) and full text (k = 0.750) screening stages.

**Figure 1.**
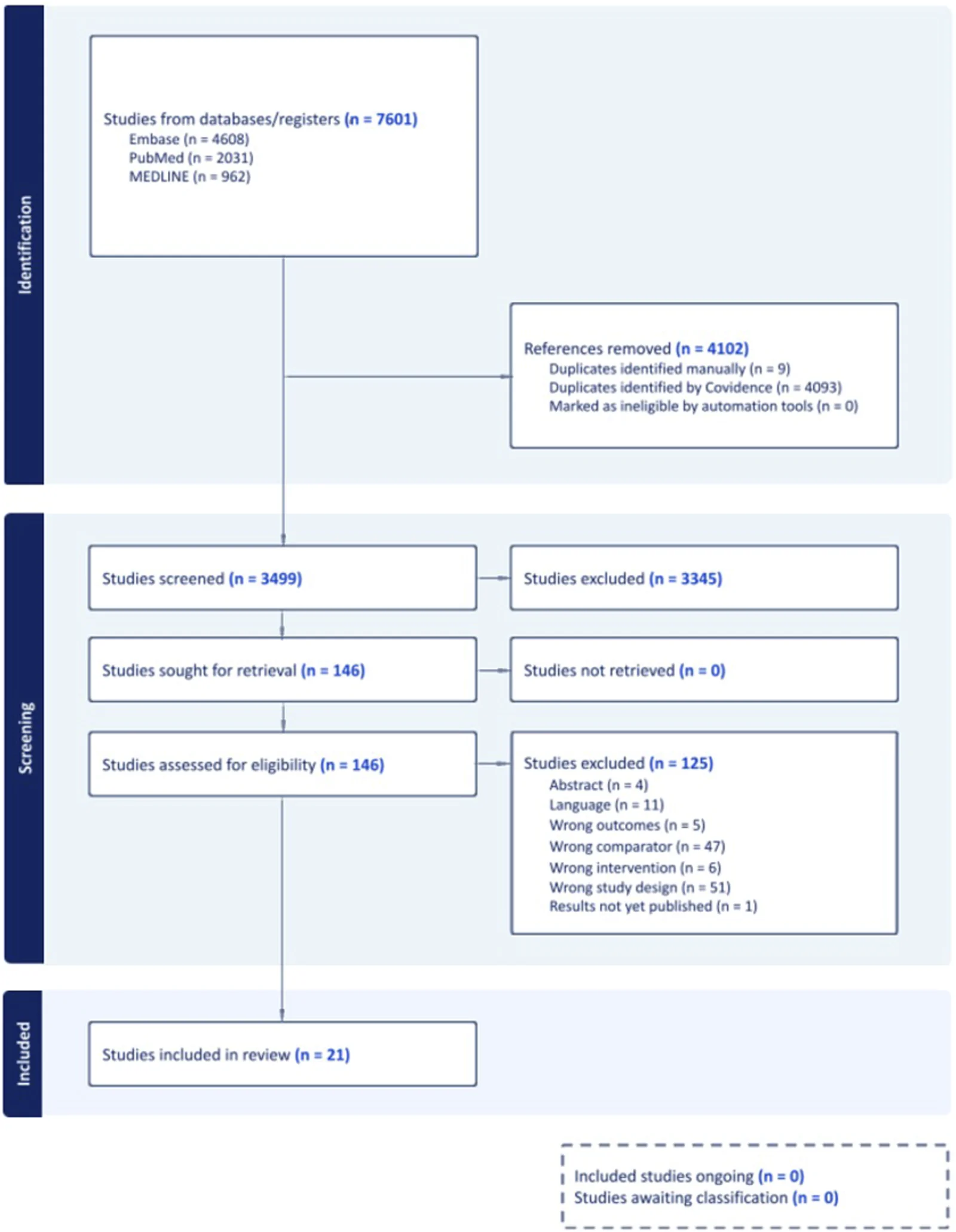
PRISMA flow diagram of study selection

### 3.2. Patient and Procedure Characteristics

Across studies, 91,285 patients were included: 8,743 (9.6%) underwent image-guided fixation (navigated or robotic) and 82,442 (90.4%) freehand fixation. Guided patients were older (50.9 vs 38.5 years), more often male (45.5% vs 40.8%), and had significantly shorter follow-up (6.36 vs 7.01 months) (all p<0.001), while BMI differences were small (29.8 vs 30.2 kg/m^2^, p=0.016) (Table 1). Navigation accounted for 93.7% and robotics for 6.3% of guided cases. This imbalance limited statistical power for robotics-specific analyses. Image guidance was used more frequently in deformity and trauma, in cervical and thoracic regions, and in multilevel constructs. Freehand fixation was predominantly used for lumbar degenerative disease. Revision procedures were more common in image-guided cohorts (1.2% vs 0.4%, p<0.001). Most surgeries in both groups were performed using open approaches (Table 2).

**Table 1.** Summary of Patient Characteristics

General Characteristics	Total	Image-guided	Freehand	p-value
Total number of patients	91,285	8,743	82,442	​
Mean age in years ± SD	40.54 n = 36,219	50.94 ± 12.12 n = 5,273	38.46 ± 10.98 n = 30,284	**<0.0001**
Sex (%)				
Male	35,749 (41.2)	2,956 (45.5)	32,746 (40.8)	**<0.00001**
Female	51,052 (58.8)	3,542 (54.5)	47,456 (59.2)	​
Mean BMI in kg/m^2^ ± SD	30.12 n = 18,302	29.80 ± 6.14 n = 1,876	30.17 ± 6.34 n = 16,326	**0.0163**
Mean follow-up time in months ± SD	6.95 ± 0.12 n = 90,496	6.36 ± 0.74 n = 8,391	7.01 ± 0.05 n = 82,005	**<0.0001**

BMI: body mass index, F: female, kg: kilogram, M: male, m: meter, SD: standard deviation.

**Table 2.** Summary of Procedure Characteristics

General Characteristics	Total	Image-guided	Freehand	p-value
Guidance (%)				NA
Navigation	7,815 (93.7)	7,815 (93.7)	NA	
Robot	522 (6.3) n = 8,337	522 (6.3) n = 8,337	NA	
Navigation System (%)				NA
O-arm-based navigation (Medtronic)	747 (39.6)	747 (39.6)	NA	
StealthStation (Medtronic)	337 (17.8)	337 (17.8)	NA	
Brainlab navigation systems	347 (18.4)	347 (18.4)	NA	
Stereotactic computer-assisted systems	456 (24.2) n = 1,887	456 (24.2) n = 1,887	NA	
Robotic System (%)				NA
Renaissance (Mazor/Medtronic)	146 (22.9)	146 (22.9)	NA	
Globus Medical systems	460 (72.2)	460 (72.2)	NA	
Chinese robotic systems	31 (4.9) n = 637	31 (4.9) n = 637	NA	
Primary Operation (%)	15,482 (99.4)	3,355 (98.8)	12,127 (99.6)	**<0.00001**
Revision Surgery (%)	86 (0.6) n = 15,568	41 (1.2) n = 3,396	45 (0.4) n = 12,172	
Region of surgery (nb of patients) (%)				
Thoracic	339 (0.5)	174 (2.8)	165 (0.2)	**<0.00001**
Lumbar	74,468 (99.5)	6,016 (96.9)	68,452 (99.8)	
Lumbosacral	34 (<0.1) n = 74,841	19 (0.3) n = 6,209	15 (<0.1) n = 68,632	
Region of surgery (nb of screws) (%)				
Cervical	291 (21.5)	124 (22.1)	169 (21.3)	**<0.00001**
Thoracic	747 (55.1)	343 (61.0)	402 (50.7)	
Lumbar	317 (23.4) n = 215	95 (16.9) n = 112	222 (28.0) n = 103	
Indication for surgery (%)				**<0.00001**
Deformity	14,537 (24.4)	2,072 (39.5)	12,460 (23.0)	
Degeneration	5,380 (9.0)	2,811 (53.6)	2,534 (4.7)	
Infection	10 (<0.1)	3 (<0.1)	4 (<0.1)	
Trauma	163 (0.3)	73 (1.4)	82 (0.1)	
Tumor	39,476 (66.3) n = 59,566*	289 (5.5) n = 5,248	39,178 (72.2) n = 54,258	
Mean number of screws	16,477 n = 2,565	5,624 n = 802	10,853 n = 1,763	0.3442
Mean number of screws per patient (SD)	5.4 (1.9, n = 1,792)	5.5 (1.8, n = 409)	5.4 (1.9, n = 1,383)	
Method (%)				0.765846
Open	3,113 (96.5)	1,213 (96.3)	1,900 (96.5)	
Percutaneous	114 (3.5) n = 3,227	46 (3.7) n = 1,259	68 (3.5) n = 1,968	
Levels (%)				
Single level	31,348 (64.8)	2,083 (44.3)	29,265 (67.0)	**<0.0001**
≥2 levels	17,045 (35.2) n = 48,393	2,617 (55.7) n = 4,700	14,428 (33.0) n = 43,693	
Mean number of levels per patient (SD)	2.5 (1.5, n = 2,762)	3.7 (2.0, n = 1,023)	1.9 (1.3, n = 1,739)	**<0.0001**

n: number of patients, NA: not applicable, nb: number, NR: not reported, SD: standard deviation; *Group-specific indication totals do not equal the overall total because one included study reported 60 patients without providing group-level allocation.

### 3.3. Pooled Outcomes (Image-guided vs Freehand)

Hardware-related revision rates were low and similar between groups (guided 1.42% vs freehand 1.35%, p=0.640). Non-hardware reoperations were less frequent with image guidance (2.44% vs 3.48%, p=0.005). URTO occurred at comparable rates (2.41% vs 2.10%, p=0.051) (Table 3).

**Table 3.** Summary of Surgical Outcomes

Outcome	Total	Image-guided	Freehand	p-value
	*n*	*n*	*n*	
Revision of hardware (%)				
Yes	707 (1.36)	88 (1.42)	619 (1.35)	0.639632
No	51,359	6,098	45,261	​
Reoperation other than revision of hardware (%)				
Yes	321 (3.14)	81 (2.44)	240 (3.48)	**0.005098**
No	9,893	3,232	6,661	​
Unexpected return to operating room (%)				
Yes	1,940 (2.13)	211 (2.41)	1,729 (2.10)	0.051444
No	89,345	8,532	80,713	​

*n*: number of patients.

### 3.4. Meta-analyses (Image-guided vs Freehand)

Random-effects meta-analysis demonstrated lower odds of hardware-related revision with image guidance (OR 0.55, 95% CI 0.40–0.75; *p*<0.001; I^2^ = 24%). No significant difference was observed for non-hardware reoperations (OR 1.03, 95% CI 0.67–1.57; *p*=0.899; I^2^ = 37%). Image guidance was associated with lower odds of URTO (OR 0.69, 95% CI 0.50–0.94; *p* = 0.018; I^2^ = 51%) (Figures 2-4: Table 4).

**Figure 2.**
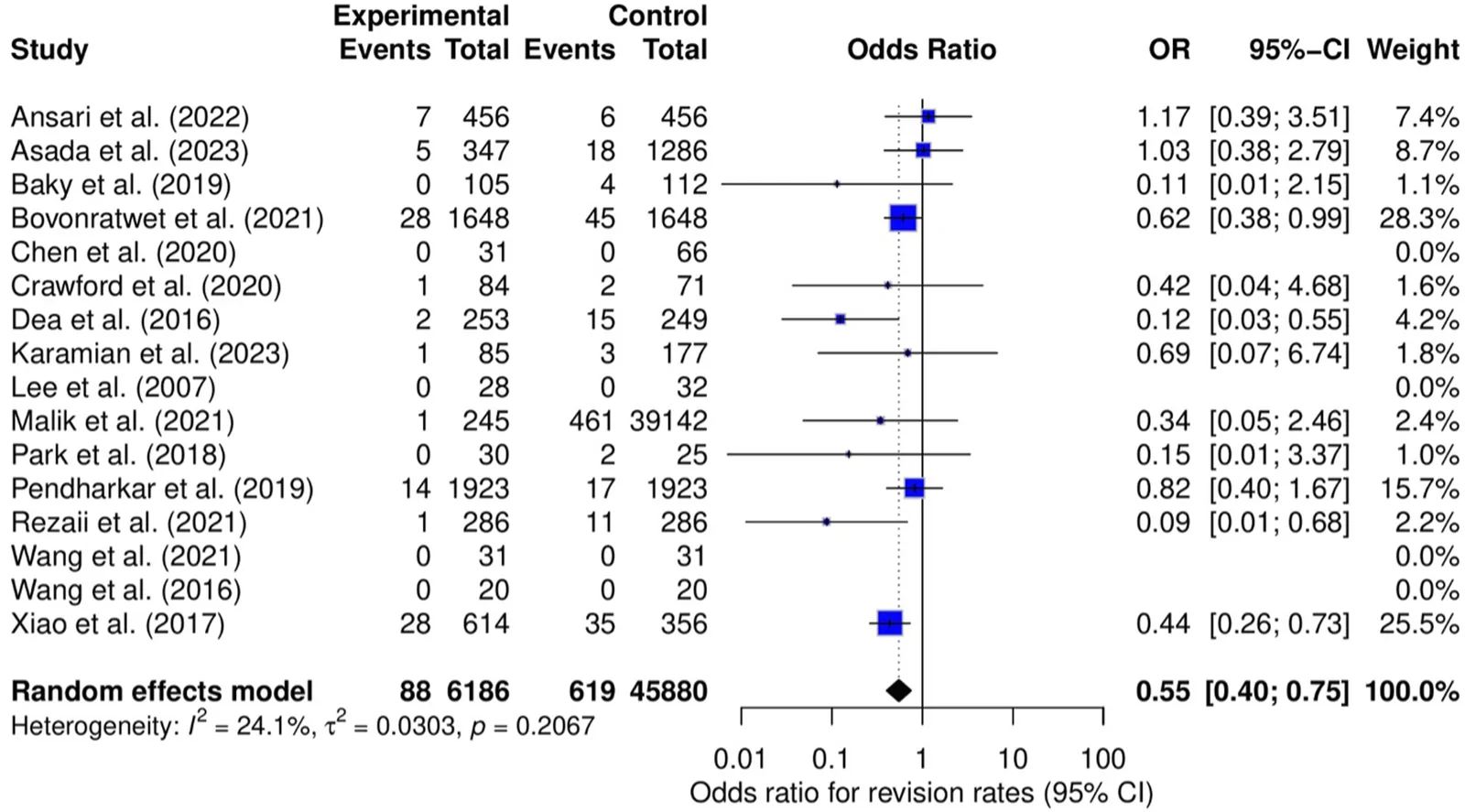
Hardware-related revision: image-guided versus freehand fixation (random-effects Mantel-Haenszel odds ratio)

**Figure 3.**
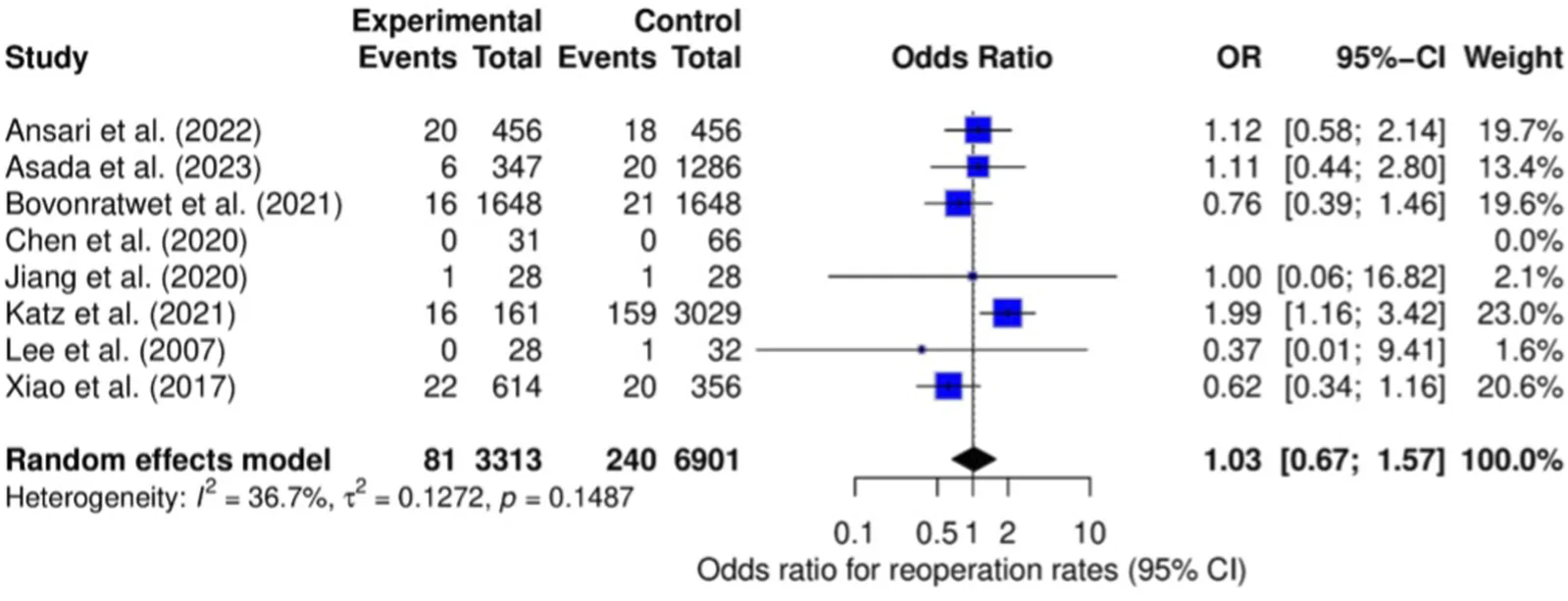
Non-hardware reoperation: image-guided versus freehand fixation (random-effects Mantel-Haenszel odds ratio)

**Figure 4.**
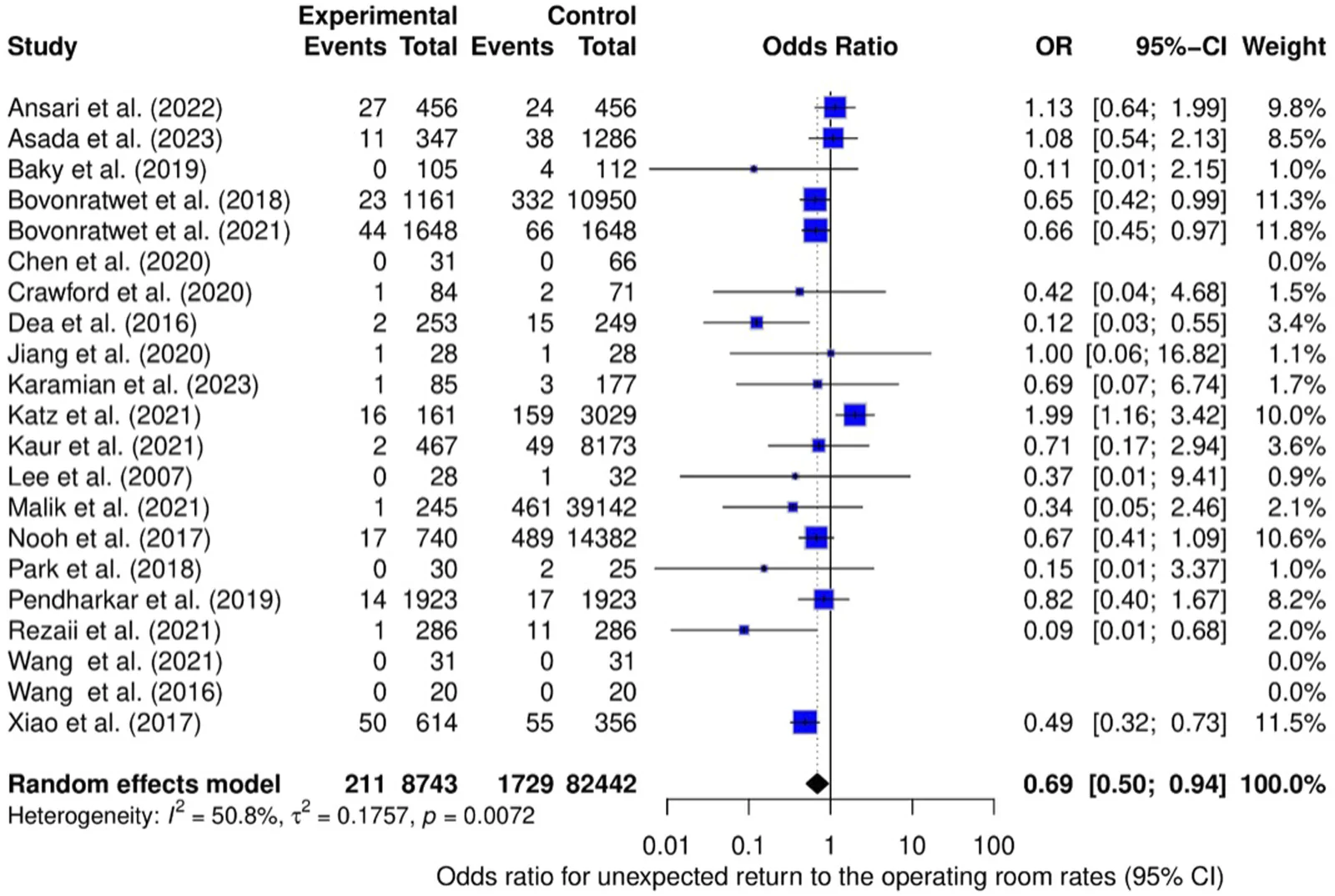
Unexpected return to the operating room: image-guided versus freehand fixation (random-effects Mantel-Haenszel odds ratio)

**Table 4.** Overall Results of Meta-analyses for Surgical Outcomes

Outcome	Nb of Studies	Total Sample (Guided/Freehand)	Pooled OR (95% CI)	p (overall effect)	I^2^ (%)	p (heterogeneity)
Revision of hardware	16	6,186/45,880	**0.55** (0.40-0.75)	**0.0002**	24.1	0.2067
Reoperation other than revision of hardware	8	3,313/6,901	**1.03** (0.67-1.57)	0.8986	36.7	0.1487
Unexpected return to operating room	21	8,743/82,442	**0.69** (0.50-0.94)	**0.0182**	50.8	**0.0072**

CI: confidence interval, nb: number, OR: odd ratio, p: p value, I^2^: inconsistency statistic.

### 3.5. Subgroup Characteristics

Navigated and robotic patients were older (49.5 and 61.8 years vs 38.5 years) and more often male than freehand patients (all p<0.001). BMI differences were small, and follow-up was longest in robotic cohorts. Navigation was used more often for deformity and trauma and in multilevel constructs (58% ≥2 levels). Robotics was used almost exclusively for lumbar degenerative disease and single-level constructs (Table 5).

**Table 5.** Summary of Patient and Procedure Characteristics for Subgroup Analysis

General Characteristics	Navigation	Robotics	Freehand	p*	p**
Total number of patients	7,785	552	82,442	​	​
Mean age in years ± SD	49.52 ± 11.86 n = 4,590	61.81 ± 12.24 n = 522	38.46 ± 10.98 n = 30,284	**<0.0001**	**<0.0001**
Sex (%)					
Male	2,544 (45.7)	265 (50.8)	32,746 (40.8)	**<0.02561**	**<0.00001**
Female	3,026 (54.3)	257 (49.2)	47,456 (59.2)		
Mean BMI in kg/m^2^ ± SD	30.32 ± 6.32 n = 1,354	28.46 ± 5.66 n = 522	30.17 ± 6.34 n = 16,326	**<0.0001**	**<0.0001**
Mean follow-up time in months ± SD	5.97 ± 0.76 n = 7,548	11.96 ± 1.23 n = 437	7.01 ± 0.05 n = 82,005	**<0.0001**	**<0.0001**
Primary Operation (%)	2,871 (98.8)	484 (98.6)	12,127 (99.6)	0.631925	**<0.00001**
Revision Surgery (%)	34 (1.2) n = 2,905	7 (1.4) n = 491	45 (0.4) n = 12.172		
Region of surgery (nb of patients) (%)	174 (3.0)	0 (0.0)	165 (0.2)	**<0.00001**	**<0.00001**
Thoracic	5,596 (96.9)	175 (94.1)	68,452 (99.8)		
Lumbar	8 (0.1)	11 (5.9)	15 (<0.1)		
Lumbosacral	n = 5,778	n = 186	n = 68,632		
Region of surgery (nb of screws) (%)	NR	291 (21.5)	169 (21.3)	NA	**<0.04820**
Cervical	NR	747 (55.1)	402 (50.7)		
Thoracic	NR	317 (23.4)	222 (28.0)		
Lumbar	​	n = 1,355	n = 103		
Indication for surgery (%)	1,911 (40.5)	0 (0.0)	12,460 (23.0)	**<0.00001**	**<0.00001**
Deformity	2,691 (57.0)	120 (100.0)	2,534 (4.7)		
Degeneration	3 (0.1)	0 (0.0)	4 (<0.1)		
Infection	73 (1.5)	0 (0.0)	82 (0.1)		
Trauma	44 (0.9)	0 (0.0)	39,178 (72.2)		
Tumor	n = 4,722	n = 120	= 54,711		
Mean number of screws	3,244	2,380	10,853	NA	0.3442
Mean number of screws per patient (SD)	N = 365	N = 437	N = 1,763		
	NR	5.4 (1.8, n = 409)	5.5 (1.9, n = 1,383)		
Method (%)					
Open	​	​	1,900 (96.5)	**<0.00001**	**<0.00001**
Percutaneous	​	​	68 (3.5) n = 1,968		
Levels (%)					
Single level	1,819 (42.0)	97 (79.5)	29,265 (67.0)	**<0.00001**	**<0.00001**
≥2 levels	2,514 (58.0) n = 4,333	25 (20.5) n = 122	14,428 (33.0) n = 43,693		
Mean number of levels per patient (SD)	4.8 (2.7, n = 614)	1.9 (0.9, n = 409)	1.9 (1.3, n = 1,739)	**<0.0001**	**<0.0001**

BMI: body mass index, F: female, kg: kilogram, M: male, m: meter, n: number of patients, NA: not applicable, nb: number, SD: standard deviation.

^*^p-value for the difference between the Robotics and Navigation groups.

^**^p-value for the difference between the Robotics, Navigation, and Freehand groups.

### 3.6. Subgroup Outcomes

Crude hardware-related revision rates were 1.50%, 1.15%, and 1.35% in the navigation, robotics, and freehand groups, respectively (p = 0.620). URTO rates were 2.32%, 2.36%, and 2.10%, respectively (p = 0.381). Non-hardware reoperation rates were 2.11%, 1.72%, and 3.48%, respectively (p < 0.001 across the three groups), with no significant difference between navigation and robotics (p = 0.608) (Table 6).

**Table 6.** Summary of Surgical Outcomes for Subgroup Analysis

Outcome	Navigation	Robotics	Freehand	p*	p**
	n	n	n		
Revision of hardware (%)					
Yes	81 (1.50)	6 (1.15)	619 (1.35)	0.523902	0.620062
No	5,336	518	45,261	​	​
Reoperation other than revision of hardware (%)					
Yes	58 (2.11)	7 (1.72)	240 (3.48)	0.607601	**0.000576**
No	2,688	399	6,661	​	​
Unexpected return to operating room (%)					
Yes	181 (2.32)	13 (2.36)	1,729 (2.10)	0.963859	0.381054
No	7,604	539	80,713	​	​

n: number of patients.

^*^p-value for the difference between Robotics and Navigation groups.

^**^p-value for the difference between Robotics, Navigation, and Freehand groups.

### 3.7. Subgroup Meta-analyses

Navigation was associated with lower odds of hardware-related revision (OR 0.51, 95% CI 0.35–0.77; p = 0.001; I^2^ = 42%) and URTO (OR 0.63, 95% CI 0.51–0.77; p < 0.001; I^2^ = 31%) compared with freehand fixation, without a significant difference in non-hardware reoperation (OR 0.79, 95% CI 0.55–1.15; p = 0.220; I^2^ = 0%). Robotics showed no significant differences versus freehand fixation for hardware-related revision (OR 0.83, 95% CI 0.35–2.00; p = 0.682; I^2^ = 0%), non-hardware reoperation (OR 1.10, 95% CI 0.46–2.64; p = 0.827; I^2^ = 0%), or URTO (OR 0.96, 95% CI 0.51–1.79; p = 0.891; I^2^ = 0%). Exploratory indirect Bucher comparisons did not identify statistically significant differences between navigation and robotics for hardware-related revision (OR 0.62, 95% CI 0.24–1.62; p = 0.328), non-hardware reoperation (OR 0.72, 95% CI 0.28–1.86; p = 0.499), or URTO (OR 0.66, 95% CI 0.34–1.27; p = 0.209); these comparisons should be interpreted cautiously because the technologies were evaluated in substantially different patient populations and procedural contexts (Figures 5-10; Tables 7-9).

**Figure 5.**
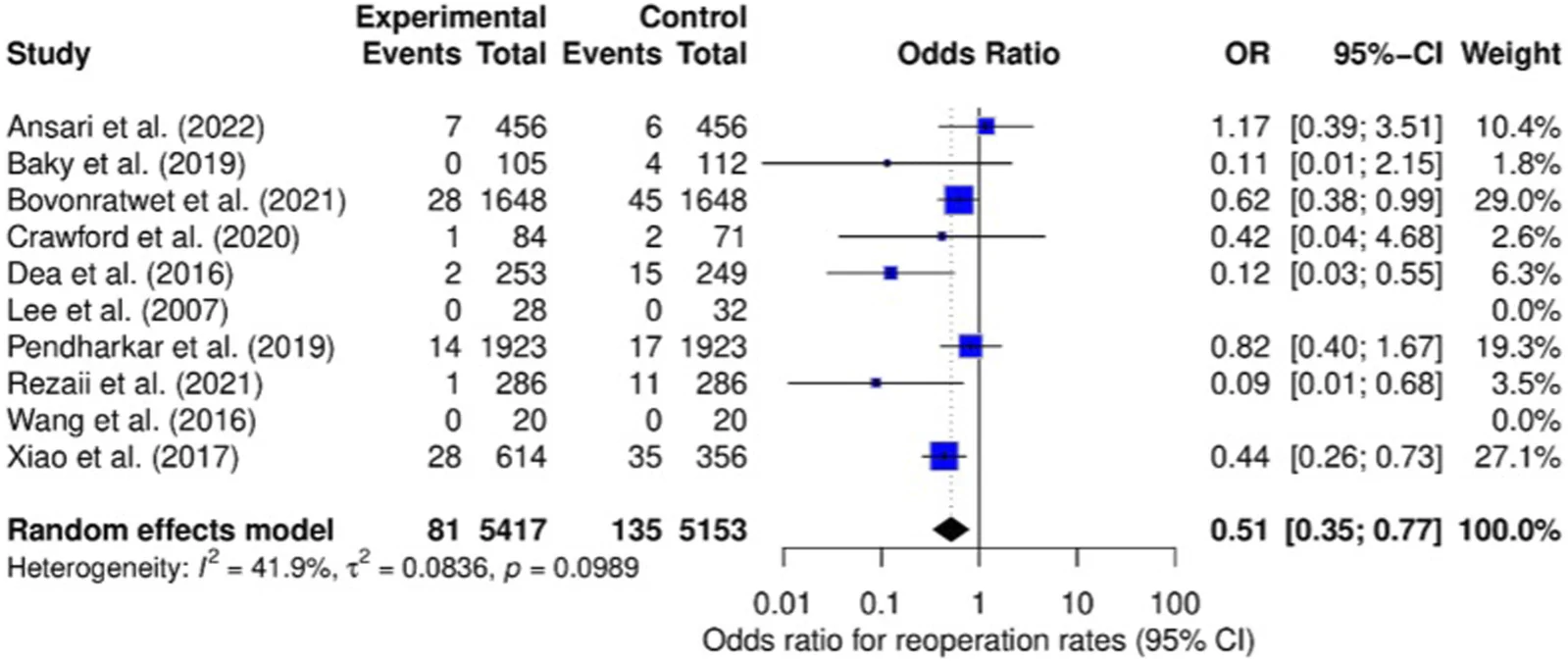
Hardware-related revision: navigation versus freehand fixation (random-effects subgroup meta-analysis)

**Figure 6.**
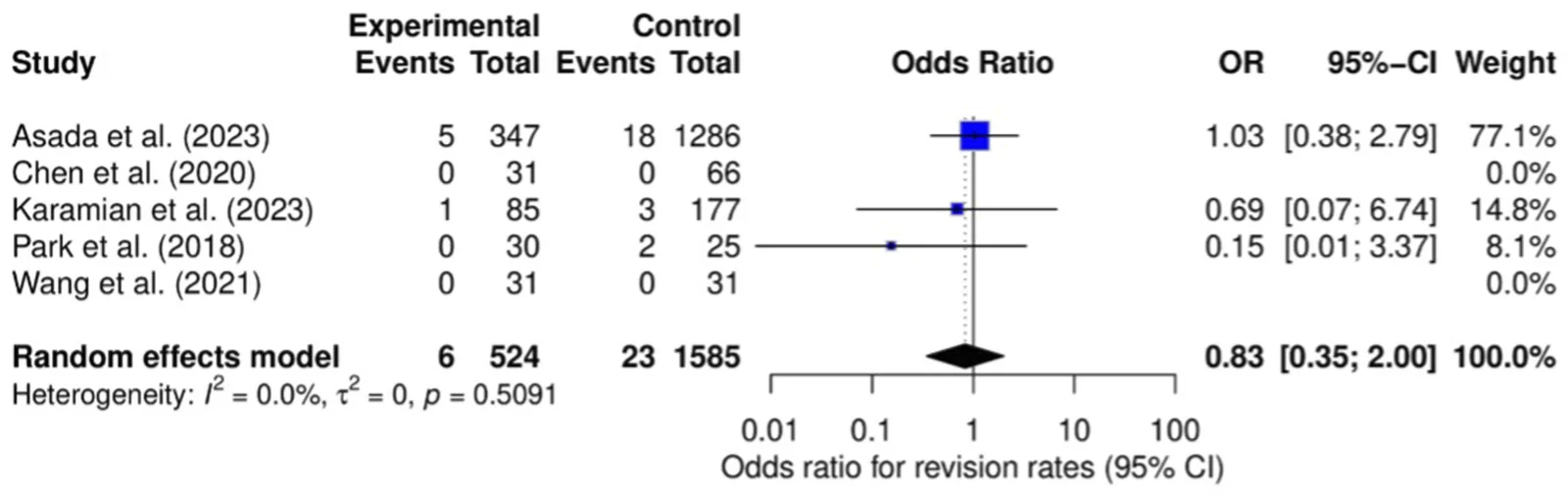
Hardware-related revision: robotics versus freehand fixation (random-effects subgroup meta-analysis)

**Figure 7.**
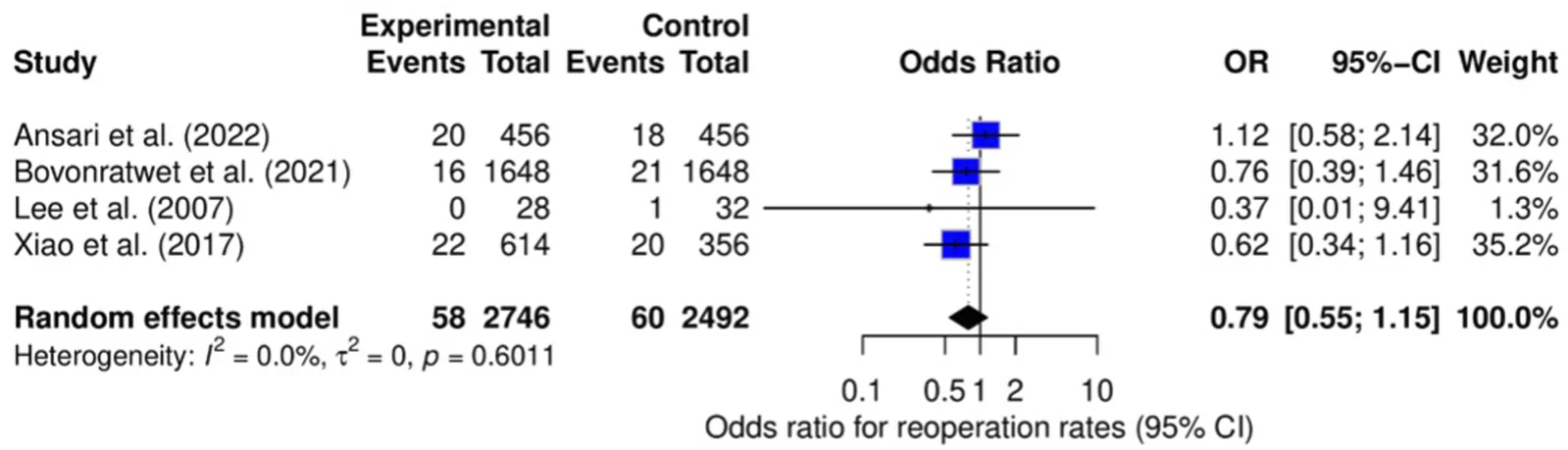
Non-hardware reoperation: navigation versus freehand fixation (random-effects subgroup meta-analysis)

**Figure 8.**
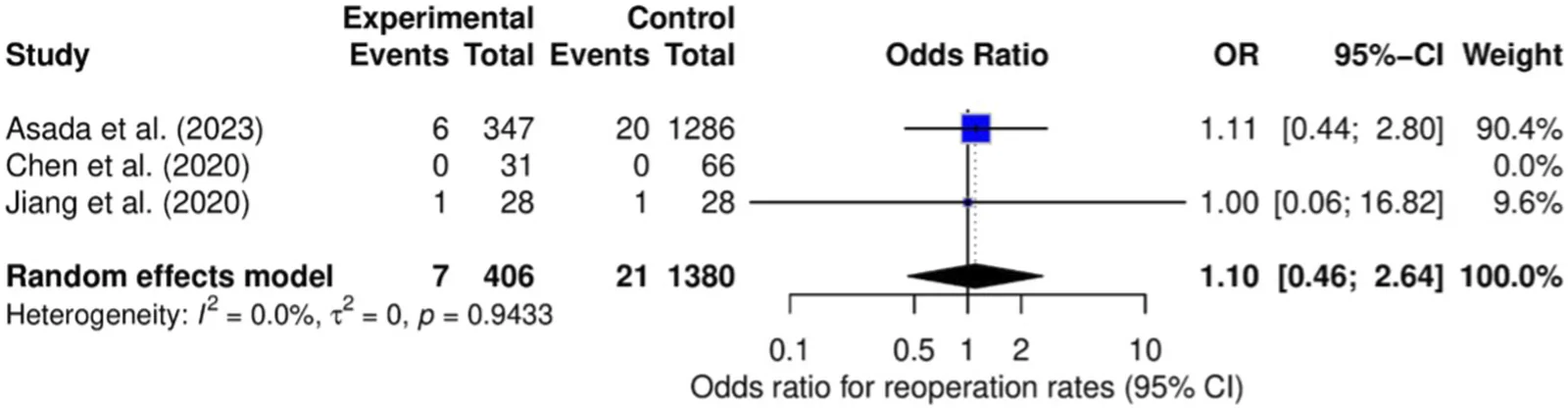
Non-hardware reoperation: robotics versus freehand fixation (random-effects subgroup meta-analysis)

**Figure 9.**
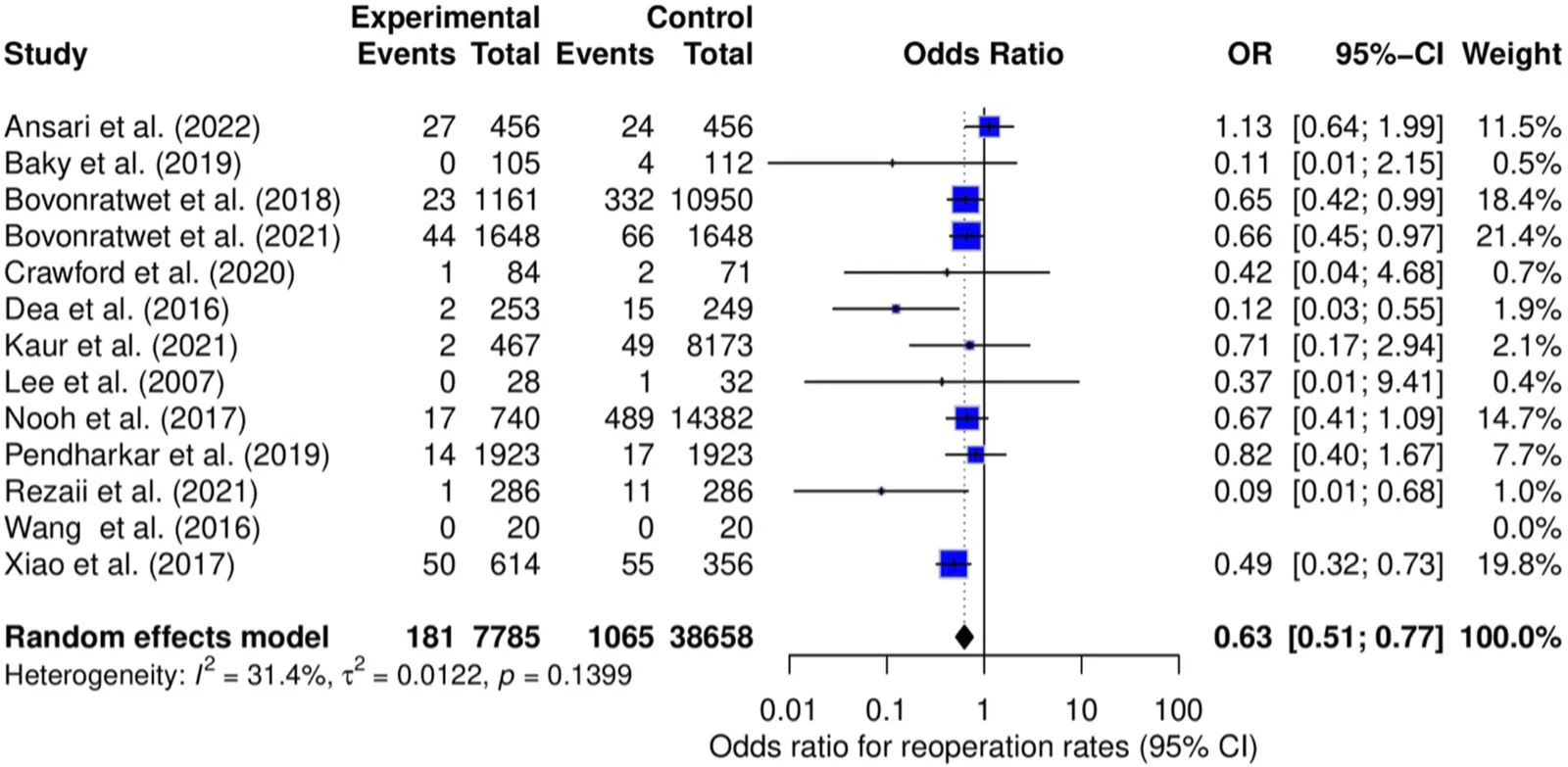
Unexpected return to the operating room: navigation versus freehand fixation (random-effects subgroup meta-analysis)

**Figure 10.**
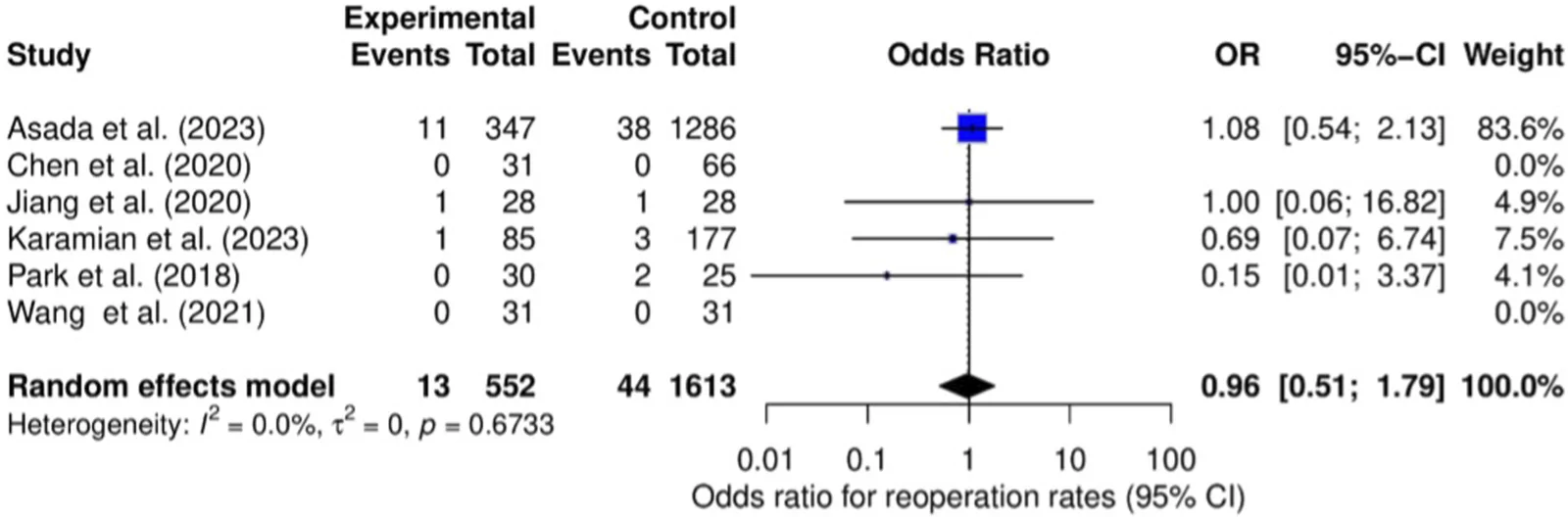
Unexpected return to the operating room: robotics versus freehand fixation (random-effects subgroup meta-analysis)

**Table 7.** Overall Results of Meta-analyses for Surgical Outcomes (Navigation vs. Freehand)

Outcome	No of Studies	Total Sample (Navigation/Freehand)	Pooled OR (95% CI)	p (overall effect)	I^2^ (%)	p (heterogeneity)
Revision of hardware	10	5,417/5,153	**0.51** (0.35-0.77)	**0.0011**	41.9	0.0989
Reoperation other than revision of hardware	4	2,746/2,492	**0.79** (0.55-1.15)	0.2197	0.0	0.6011
Unexpected return to operating room	13	7,785/38,658	**0.63** (0.51-0.77)	**<0.0001**	31.4	0.1399

CI: confidence interval, nb: number, OR: odd ratio, p: p value, I^2^: inconsistency statistic.

**Table 8.** Overall Results of Meta-analyses for Surgical Outcomes (Robotics vs. Freehand)

Outcome	Nb of Studies	Total Sample (Robotics/Freehand)	Pooled OR (95% CI)	p (overall effect)	I^2^ (%)	p (heterogeneity)
Revision of hardware	5	524/1,585	**0.83** (0.35-2.00)	0.6821	0.0	0.5091
Reoperation other than revision of hardware	3	406/1,380	**1.10** (0.46-2.64)	0.8272	0.0	0.9433
Unexpected return to operating room	6	552/1,613	**0.96** (0.51-1.79)	0.8911	0.0	**0.6733**

CI: confidence interval, nb: number, OR: odd ratio, p: p value, I^2^: inconsistency statistic.

**Table 9.** Overall Results of Meta-analyses for Surgical Outcomes (Navigation vs. Robotics)

Outcome	No of Studies (Navigation/Robotics)	Total Sample (Navigation/Robotics)	Pooled OR (95% CI)	p-value
Revision of hardware	10/5	5,417/524	**0.618** (0.236-1.620)	0.3280
Reoperation other than revision of hardware	4/3	2,746/406	**0.721** (0.279-1.861)	0.4986
Unexpected return to operating room	13/6	7,785/552	**0.657** (0.341-1.266)	0.2090

CI: confidence interval, nb: number, OR: odd ratio.

### 3.8. Risk of Bias

Overall risk of bias was high, driven by retrospective study designs, confounding, and incomplete outcome reporting, which limited causal inference. The two randomized trials were judged to have some concerns or a high risk of bias, and most nonrandomized studies were judged to be at serious risk of bias using ROBINS-I (Supplemental Table S2). For the URTO outcome, Egger’s test was not significant (t = -1.30, df = 16, p=0.21), with no clear funnel plot asymmetry observed (Figure 11).

**Figure 11.**
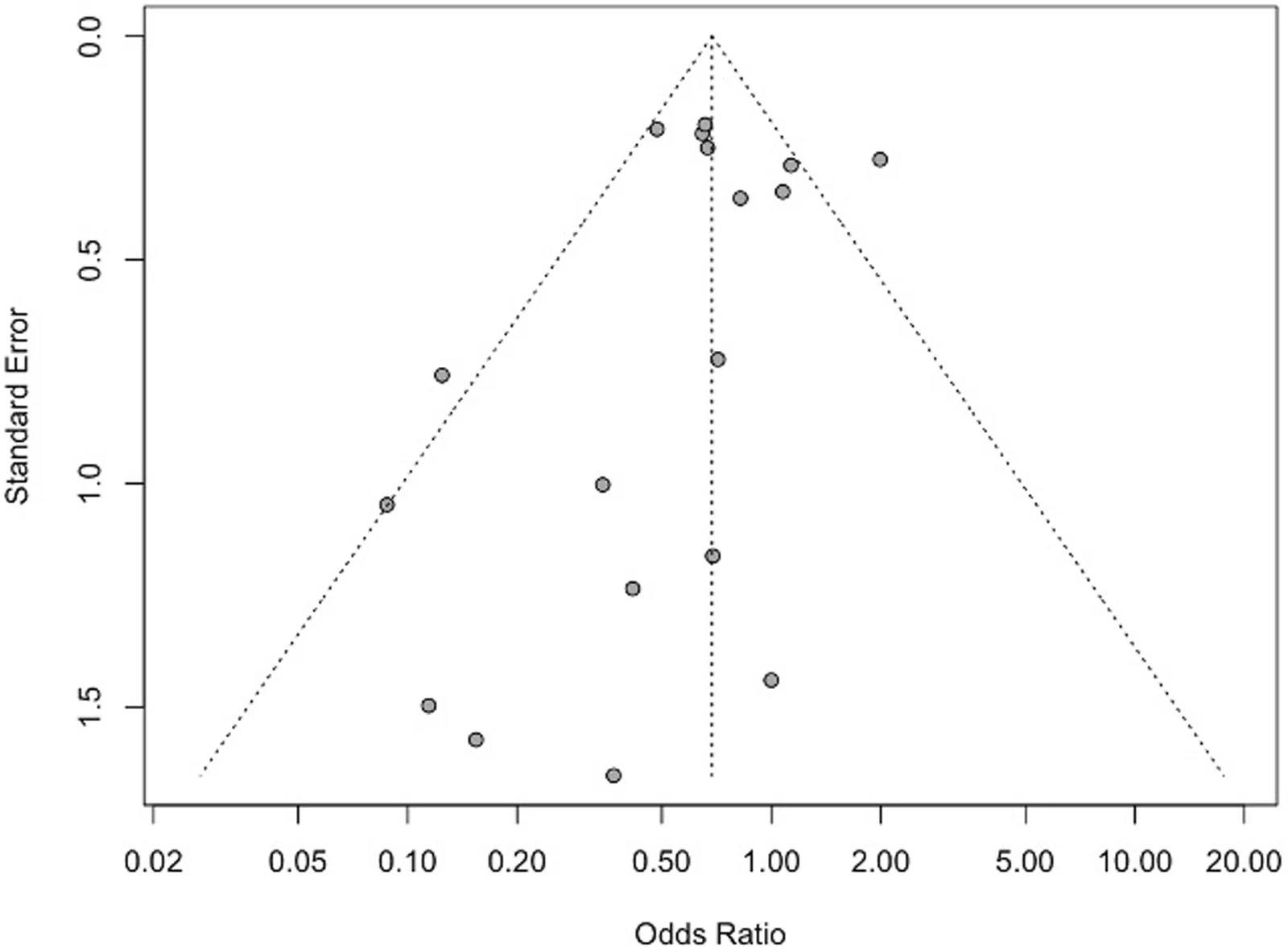
Funnel plot assessing publication bias for unexpected return to the operating room

## 4. Discussion

This systematic review and meta-analysis evaluated navigation, robotics, and freehand screw fixation across more than 91,000 patients, with a focus on URTO as a patient-centered outcome rather than screw-placement accuracy alone. The crude aggregate event rates in Tables 3 and 6 did not consistently favour image guidance, whereas the random-effects meta-analysis showed lower odds of hardware-related revision and URTO. This apparent discrepancy reflects the different analytic approaches: crude rates pool all patients without study-level weighting, whereas the meta-analysis combines study-specific odds ratios using random-effects weighting and incorporates between-study heterogeneity. The pooled estimates nevertheless remain susceptible to residual confounding because most included studies were retrospective and the cohorts differed substantially in baseline characteristics and case complexity. Robotics showed no statistically significant differences compared with freehand fixation, but its underrepresentation and distinct case mix preclude conclusions of equivalence.

URTO represents a valuable patient-centered endpoint that captures the net clinical effect of navigation, integrating both hardware-related failures and broader postoperative complications. Prior work has demonstrated that navigation improves screw placement and reduces revision for malpositioned hardware.^9,10,34^ In the present meta-analysis, navigation was associated with lower odds of hardware-related revision and URTO, with no significant difference in non-hardware reoperation. This pattern is consistent with the mechanism of navigation: it improves technical accuracy but does not directly influence complications such as infection, hematoma, pseudarthrosis, or medical events.

Demographic and procedural differences across groups help explain these results. Navigation was more frequently used in older patients, deformity and trauma indications, thoracic and cervical regions, and multilevel constructs—situations with intrinsically higher risk. Freehand fixation was most common for lumbar degenerative disease and malignancy, often involving shorter constructs. Robotics appeared to be deployed in a narrower clinical context, largely degenerative lumbar disease and single-level instrumentation. These case-mix differences likely contributed to the higher unadjusted complication rates observed in image-guided cohorts. Although the study-level meta-analysis favoured navigation for hardware-related revision and URTO, it could not fully adjust for patient-level differences in age, surgical indication, construct length, or procedural complexity. Residual confounding and selection bias therefore remain, and the observed associations should not be interpreted as definitive evidence of a causal protective effect.

The potential benefits of navigation must be interpreted alongside its trade-offs. Navigation requires increased operating room traffic, more equipment, and workflow-related setup which raises the concern for sterile field contamination. Additionally, Katz et al reported higher unadjusted reoperation rates in adult spinal deformity navigated cases (9.9% vs 5.2%, p=0.011), likely reflecting longer operative time in navigated cohorts (405 min vs 320 min, p<0.001), increased transfusion requirements in navigated cohorts (52.2% vs 41.8%, p=0.01), and greater procedural complexity rather than a direct causal effect of navigation itself.^12^ Similarly, concerns have been raised regarding infection risk. Berman et al observed higher infection-related return to the operating room in patients undergoing intraoperative 3D navigation (4.9% vs 0.6%, p=0.025), although this association did not reach statistical significance after adjustment (adjusted OR 6.49, p=0.073).^11^ In contrast, Shuman et al found that navigation was associated with decreased odds of return to the operating room for wound-related procedures despite longer operative times, highlighting the inconsistency in current literature.^35^ Taken together, these findings provide no clear signal of increased non-hardware reoperation with navigation. However, heterogeneity and residual confounding preclude definitive conclusions regarding infection or other non-hardware complications.

Economic considerations may also influence the value of navigation.^21^ Although navigation entails substantial acquisition and operating costs, avoidance of hardware-related revision could offset some of these expenses; however, formal cost-effectiveness analysis was beyond the scope of this review.

This study has several important limitations. The evidence base was predominantly retrospective, with inherent risks of selection bias, residual confounding, and inconsistent outcome reporting, limiting causal inference. Heterogeneity in study design, outcome definitions, and reporting formats—including per-patient versus per-screw analyses—further reduced comparability across studies. Mean follow-up was relatively short in both groups—6.36 months for image-guided fixation and 7.01 months for freehand fixation—which may have undercaptured reoperations occurring beyond the early postoperative period. Robotics was substantially underrepresented, limiting statistical power and restricting conclusions regarding its comparative effectiveness. Moreover, navigation and robotics were evaluated in markedly different patient populations and surgical indications, and the absence of direct head-to-head comparisons required exploratory indirect analyses that remain susceptible to confounding. Future prospective, multicenter studies with standardized outcome definitions, adequate follow-up, and direct platform comparisons are needed to clarify the role of image guidance in reducing URTO.^36^

## 5. Conclusion

Navigation was associated with lower odds of hardware-related revision and a modest reduction in URTO, without a difference in non-hardware reoperation. These associations should be interpreted cautiously because the evidence was predominantly retrospective and remained subject to residual confounding. Robotics showed no statistically significant advantage over freehand fixation, but its underrepresentation and differing case mix preclude definitive conclusions regarding comparative effectiveness. Prospective, multicenter studies with standardized outcome definitions, adequate follow-up, and direct platform comparisons are needed.

## Supplemental Material

Supplemental material - The Impact of Image-Guided Screw Placement on Unexpected Return to the Operating Room: A Systematic Review and Meta-Analysis of 91,285 Patients Supplemental material for The Impact of Image-Guided Screw Placement on Unexpected Return to the Operating Room: A Systematic Review and Meta-Analysis of 91,285 Patients by Jaskaran Singh, Marion Sylvain, Mikko Vorster, Bona Baissa, Aazad Abbas, and Michael Goytan in Global Spine Journal.

Supplemental material - The Impact of Image-Guided Screw Placement on Unexpected Return to the Operating Room: A Systematic Review and Meta-Analysis of 91,285 Patients Supplemental material for The Impact of Image-Guided Screw Placement on Unexpected Return to the Operating Room: A Systematic Review and Meta-Analysis of 91,285 Patients by Jaskaran Singh, Marion Sylvain, Mikko Vorster, Bona Baissa, Aazad Abbas, and Michael Goytan in Global Spine Journal.
