# Supplementary material for: The Impact of Image-Guided Screw Placement on Unexpected Return to the Operating Room: A Systematic Review and Meta-Analysis of 91,285 Patients
Source: Global Spine J. 2026 Sep 28:21925682261493032. Online ahead of print. doi: 10.1177/21925682261493032 (PMC13619974; doi:10.1177/21925682261493032)
Supplement: Supplementary file 1 [file sj-pdf-1-gsj-10.1177_21925682261493032.pdf]

Supplemental Table S1. Study Characteristics

Author	Article Title	Year	Country	Funding	Methodology	Evidence
**Ansari et al.**	A Retrospective Comparative Analysis of Perioperative Complications in Navigated versus Conventional Thoracolumbar Fusion in the Setting of Adult Spinal Deformity	2022	USA	None	RS	2b
**Asada et al.**	Robot-navigated pedicle screw insertion can reduce intraoperative blood loss and length of hospital stay: analysis of 1,633 patients utilizing propensity score matching	2023	USA	None	RS	2b
**Baky et al.**	Intraoperative Computed Tomography-Guided Navigation for Pediatric Spine Patients Reduced Return to Operating Room for Screw Malposition Compared With Freehand/Fluoroscopic Techniques	2019	USA	National Institute of Arthritis and Musculoskeletal and Skin Diseases, NIH T32 Musculoskeletal Training grant	RS	2b
**Bovonratwet et al.**	Comparison of 30-Day Complications Between Navigated and Conventional Single-level Instrumented Posterior Lumbar Fusion	2018	USA	None	RS	2b (3)
**Bovonratwet et al.**	Computer-Assisted Navigation Is Associated With Decreased Rates of Hardware-Related Revision After Instrumented Posterior Lumbar Fusion	2021	USA	None	RS	2b
**Chen et al.**	Robot-assisted orthopedic surgery in the treatment of	2020	China	Shanghai Jiao	RS	2b (3)

	adult degenerative scoliosis: a preliminary clinical report			Tong University, Municipal Science and Technology Committee, and Shen-Kang Hospital Development Center		
**Crawford et al.**	Transpedicular Screw Placement Accuracy Using the O-Arm Versus Freehand Technique at a Single Institution	2020	USA	None	RS	2b
**Dea et al.**	Economic evaluation comparing intraoperative cone beam CT-based navigation and conventional fluoroscopy for the placement of spinal pedicle screws: a patient-level data cost-effectiveness analysis	2016	Canada	NR	RS	2b
**Jiang et al.**	Robot-Assisted versus Freehand Instrumentation in Short-Segment Lumbar Fusion: Experience with Real-Time Image-Guided Spinal Robot	2020	USA	NR	RS	2b
**Karamian et al.**	Clinical Outcomes of Robotic Versus Freehand Pedicle Screw Placement After One-to Three-Level Lumbar Fusion	2023	USA	None	RS	2b
**Katz et al.**	Impact of Navigation on 30-Day Outcomes for Adult Spinal Deformity Surgery	2021	USA	None	RS	2b
**Kaur et al.**	Does Navigation Make Spinal Fusion for Adolescent Idiopathic Scoliosis Safer? Insights From a National Database	2021	USA	None	RS	2b (3)

**Lee et al.**	Clinical Accuracy of Cervicothoracic Pedicle Screw Placement	2007	Canada	None	RS	2b
**Malik et al.**	Robotic-Assisted Versus Conventional Posterior Lumbar Fusion—An Analysis of 90-Day Complications and Readmissions	2021	USA	NR	RS	2b
**Nooh et al.**	Use of Computer Assistance in Lumbar Fusion Surgery: Analysis of 15 222 Patients in the ACS-NSQIP Database	2017	USA	None	RS	2b
**Park et al.**	Radiographic and Clinical Outcomes of Robot-Assisted Posterior Pedicle Screw Fixation: Two-Year Results from a Randomized Controlled Trial	2018	South Korea	Basic Science Research Program (National Research Foundation of Korea), Ministry of Science and ICT, Mazor Robotics Inc. and Medtronic Inc.	RCT	1b
**Pendharkar et al.**	Conventional Versus Stereotactic Image-guided Pedicle Screw Placement During Posterior Lumbar Fusions	2019	USA	None	RS	2b (3)
**Rezaii et al.**	Conventional Versus Stereotactic Image Guided Pedicle Screw Placement During Spinal Deformity Correction: A Retrospective Propensity Score-Matched Study of a National Longitudinal Database	2021	USA	NR	RS	2b
**Wang et al.**	Accuracy and deviation analysis of robot- assisted spinal implants: A retrospective overview of 105 cases and preliminary comparison to open freehand surgery in	2021	China	China Postdoctoral Science Foundation,	RS	2b

	lumbar spondylolisthesis			National Natural Science Foundation of China		
**Wang et al.**	Navigation Makes Transforaminal Lumbar Interbody Fusion Less Invasive	2016	China	NR	RCT	1b
**Xiao et al.**	Clinical outcomes following spinal fusion using an intraoperative computed tomographic 3D imaging system	2017	USA	NR	RS	2b

NR: Not Reported, RCT: randomized controlled trial, RS: retrospective study, USA: United States of America
