# Supplementary material for: The Impact of Image-Guided Screw Placement on Unexpected Return to the Operating Room: A Systematic Review and Meta-Analysis of 91,285 Patients
Source: Global Spine J. 2026 Sep 28:21925682261493032. Online ahead of print. doi: 10.1177/21925682261493032 (PMC13619974; doi:10.1177/21925682261493032)
Supplement: Supplementary file 2 [file sj-pdf-2-gsj-10.1177_21925682261493032.pdf]

Supplemental Table S2.

Table A.
RoB 2 – Risk of Bias for Randomized Controlled Trials

Study	Randomization process	Deviations from intended interventions	Missing outcome data	Measurement of the outcome	Selection of reported results	Overall risk of bias
Park et al. (2018)	Some concerns	Some concerns	High risk	Some concerns	Low risk / Some concerns	High risk
Wang et al. (2016)	Some concerns	Some concerns	Low risk	Low risk / some concerns	Low risk	Some concerns

Table B.
ROBINS-I Risk of Bias Assessment for Included Studies

Study	Confounding	Selection of participants	Classification of interventions	Deviations from intended interventions	Missing data	Measurement of outcomes	Selection of the reported results	Overall
Ansari et al. (2022)	Serious	Moderate	Moderate	Low	Moderate	Low	Moderate	Serious
Asada et al. (2023)	Serious	Moderate	Moderate	Low	Moderate	Low	Moderate	Serious
Baky et al. (2019)	Serious	Moderate	Moderate	Low	Moderate	Low	Moderate	Serious
Bovonratwet et al. (2018)	Moderate	Moderate	Low	Low	Low	Moderate	Low	Moderate
Bovonratwet et al. (2021)	Moderate	Serious	Moderate	Low	Low	Moderate	Low	Serious
Chen et al. (2020)	Serious	Moderate	Moderate	Low	Moderate	Low	Moderate	Serious
Crawford et al. (2020)	Serious	Moderate	Moderate	Low	Moderate	Low	Moderate	Serious
Dea et al. (2016)	Serious	Moderate	Moderate	Low	Moderate	Low	Moderate	Serious
Jiang et al. (2020)	Serious	Moderate	Moderate	Low	Moderate	Low	Moderate	Serious
Karamian et al. (2023)	Serious	Moderate	Moderate	Low	Moderate	Low	Moderate	Serious
Katz et al. (2021)	Serious	Serious	Moderate	Low	Serious	Low	Moderate	Serious
Kaur et al. (2021)	Serious	Moderate	Moderate	Low	Moderate	Low	Moderate	Serious
Lee et al. (2007)	Serious	Moderate	Moderate	Low	Low	Low	Low	Serious
Malik et al. (2021)	Serious	Moderate	Moderate	Low	Serious	Low	Moderate	Serious
Nooh et al. (2017)	Serious	Moderate	Moderate	Low	Serious	Low	Moderate	Serious
Pendharkar et al. (2019)	Serious	Moderate	Moderate	Low	Moderate	Low	Moderate	Serious
Rezaii et al. (2021)	Serious	Moderate	Moderate	Low	Moderate	Low	Moderate	Serious
Wang et al. (2021)	Serious	Moderate	Moderate	Low	Moderate	Low	Moderate	Serious
Xiao et al. (2017)	Serious	Moderate	Moderate	Low	Moderate	Low	Low	Serious
